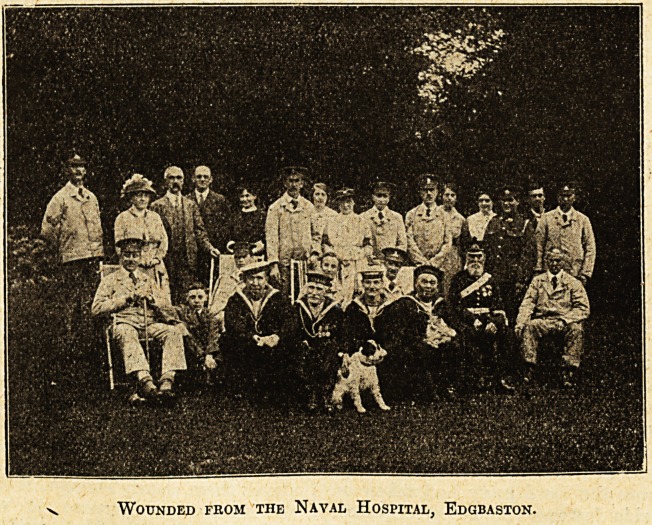# Lessening the Tedium of Hospital Life

**Published:** 1916-10-28

**Authors:** 


### LESSENING THE TEDIUM OF HOSPITAL LIFE.

### The Birmingham System at Work.

THERE has been previous allusion in THE HOSPITAL to the efforts made by the Birmingham branches of the British Red Cross Society and the Navy League for the entertainment of the wounded in the hospitals of the city and district. Since these have been successful enough to lead to an extension of the area thus provided for by the inclusion of expeditions to Wales and elsewhere, a review of the work accomplished is of interest.

The entertainments have consisted of visits to the theatres, garden parties to public and private grounds, and what may be termed tours by travelling companies to the wounded in different hospitals. By these means some 28,000 men have visited the theatres of the city and district, while over 10,000 have been entertained at garden parties. Besides this, 25,000 men have had the benefit of concerts and general entertainments, over 200 of which have been given at the hospitals. In addition, drives into the country have been arranged several times a week for men who were able to enjoy them, and many well-known people have given the garden parties of which mention has been made above.

The accompanying illustration is a pleasant record of one of these afternoons. The original photograph here reproduced was taken by Mr. Arthur J. Leeson on the afternoon when the wounded from the Naval Hospital, Edgbaston, Birmingham, visited Leaholme, Barnt Green, Worcestershire, on the kind invitation of Mr. and Mrs. Arthur J. Leeson. Mr. Leeson is vice-chairman and honorary treasurer of the Birmingham branch of the Navy League, and is seen in the photograph with Mrs. Leeson and their daughters. It will be noticed that the party includes several naval and military veterans who fought in the Crimea and in the Mutiny.

WOUNDED FROM THE NAVAL HOSPITAL, EDGBASTON.

The system of entertainments, one of which is here pleasantly illustrated, has won the approval not only of the men but of the authorities, and Sir Alfred Keogh, Director - General of the Medical Services; Lieutenant-General H. C. Sclater, G.C.B., Commander - in - Chief Southern Command; and the Secretary of State for War have written to say how valuable the work is to the men and the staff in the hospitals. The centre of the organisation is Queen's College, in Paradise Street, where the organising secretary is installed, and would, no doubt, give practical hints to any inquirers on the methods adopted to make this system of entertainments a workable success.

## Figures and Tables

**Figure f1:**